# The Role of Extracorporeal Cardiopulmonary Resuscitation in Pediatric Intraoperative Cardiac Arrest

**DOI:** 10.7759/cureus.59940

**Published:** 2024-05-09

**Authors:** Carolina Salgueirinho, André Correia, Inês Graça, Raquel Oliveira, José Dias

**Affiliations:** 1 Department of Anesthesiology, São João University Hospital Center, Porto, PRT

**Keywords:** pediatric intraoperative cardiac arrest, cardiopulmonary resuscitation, intraoperative complications, extracorporeal circulation, cardiac arrest

## Abstract

Refractory pediatric intraoperative cardiac arrest is a rare but challenging situation for the anesthesiologist. This case describes an intraoperative extracorporeal cardiopulmonary resuscitation (ECPR) in a 16-year-old male who suffered a sudden cardiac arrest during elective thoracolumbar stabilization. The patient recovered to his pre-operative baseline without any neurological sequela secondary to cardiac arrest. Good quality of conventional resuscitation measures, prompt activation of the extracorporeal membrane oxygenation (ECMO) team, and a multidisciplinary coordinated approach were key factors in ECPR success. Despite the lack of robust evidence in pediatrics, case reports like ours outline the life-saving potential of intraoperative ECPR in refractory cardiac arrest scenarios.

## Introduction

The survival rates of pediatric patients with cardiac arrest with good neurological outcomes are intrinsically related to the efficient and timely application of cardiopulmonary resuscitation (CPR) [1]. Despite adequate conventional resuscitation measures, refractory intra-operative cardiac arrest remains a challenging issue in anesthesia.

Extracorporeal membrane oxygenation (ECMO) combined with CPR, also known as extracorporeal cardiopulmonary resuscitation (ECPR), has become a lifesaving approach in cases of refractory cardiac arrest to conventional measures [2,3]. ECPR provides support for circulation and oxygenation while investigations and treatment of the underlying etiology are being carried out [2,4]. In this sense, the Extracorporeal Life Support Organization (ELSO), in 2021, published guidelines related to the application of ECPR in pediatric patients. It was reported to have better outcomes in the pediatric population compared to adults [2].

We present a case report that supports the ECPR in a case of pediatric refractory in-hospital cardiac arrest, its role in good neurological outcomes, and the need for the establishment of local protocols.

This is a case report from a University Hospital, which is a high-volume ECMO center (80-100 patients/year) [5].

Written consent has been obtained from the patient’s mother. This article was previously presented as a meeting abstract at the 2022 Annual Meeting of the European Society for Paediatric Anaesthesiology that took place from September 29, 2022, to October 1, 2022, at the Centro de Congressos de Lisboa, Lisbon, Portugal.

## Case presentation

**Figure 1:**
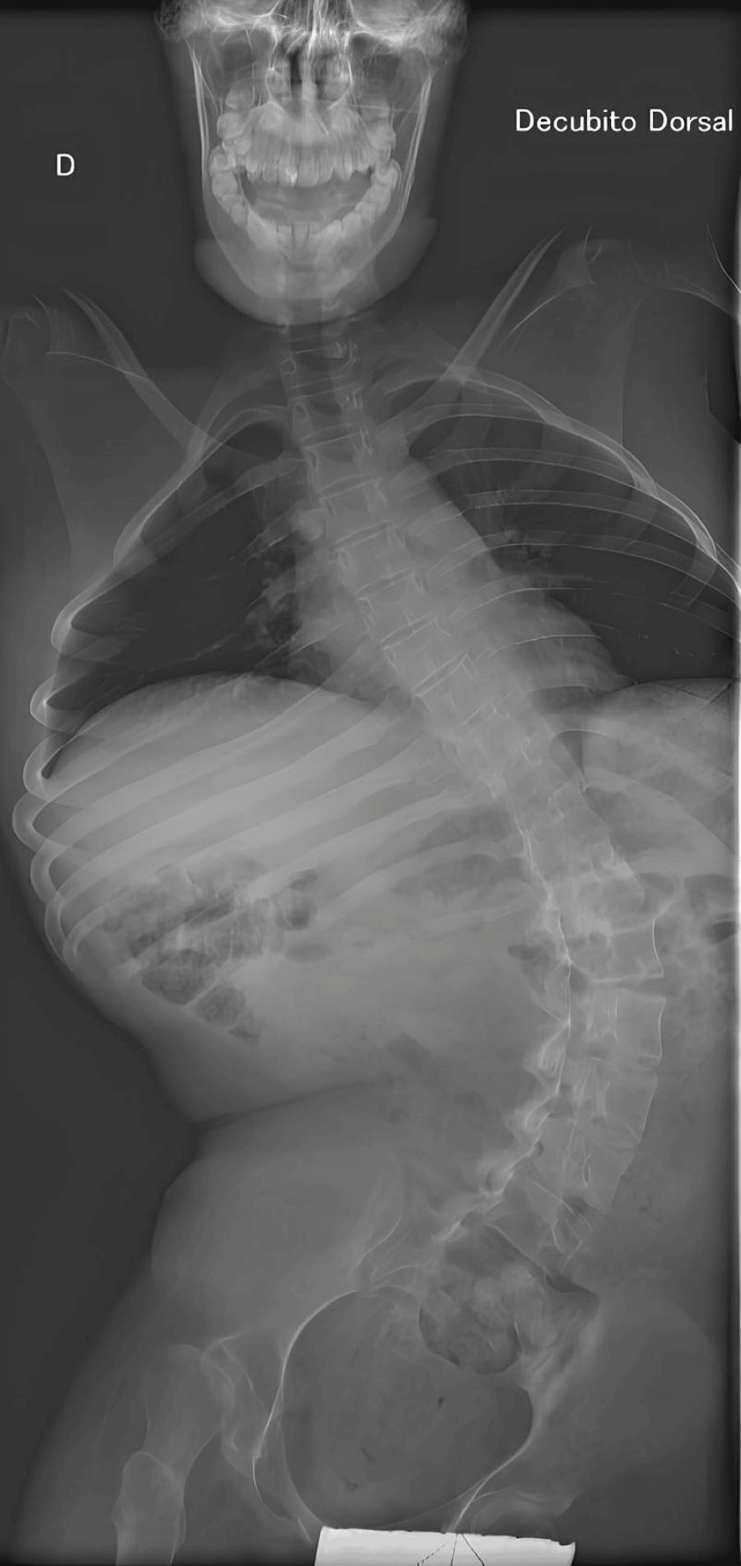
Pre-operative X-ray Moderate thoracolumbar scoliosis (Cobb angle 35.6º); Decubito dorsal - supine

A 16-year-old male was admitted for elective thoracolumbar stabilization. He presented a medical history of Duchenne muscular dystrophy diagnosed by genetic testing and moderate obstructive sleep apnea under nocturnal non-invasive ventilation (American Society of Anaesthesiologists (ASA) physical status class III). The chest X-ray was unremarkable, apart from the scoliosis (Figure 1).

**Figure 2:**
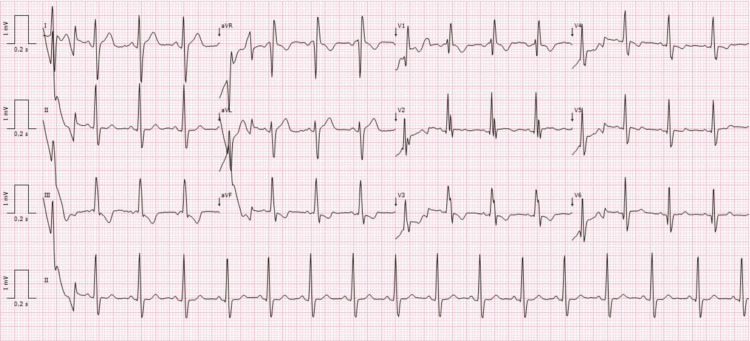
Pre-operative electrocardiogram Sinus rhythm, pulse rate of 98 beats per minute, right bundle branch block, QRS duration 110 ms, PR interval 128 ms.

The electrocardiogram exhibited a sinus rhythm, a pulse rate of 98 beats per minute, and a right bundle branch block (Figure 2).

Pre-operative transthoracic echocardiography showed no structural abnormalities and good biventricular function. The complete blood count, prothrombin and activated partial thromboplastin time, renal function, and electrolytes were within the normal range, while creatine phosphokinase was elevated at 1149 U/l. Previous surgical interventions occurred without any anesthetic complications.

On arrival in the operating room, ASA monitoring standards I and II were established, anesthesia was induced with propofol and fentanyl intravenous boluses and muscle paralysis was obtained with rocuronium bromide. Orotracheal intubation was performed without intercurrences. The patient was then positioned in a prone position and anesthesia was maintained using a target-controlled infusion with propofol and remifentanil, titrated to an adequate depth of anesthesia. Additional monitoring included invasive arterial blood pressure, urine output, somatosensory, and motor-evoked potentials.

The intraoperative course was uneventful until two hours after the incision, when end-tidal carbon dioxide decreased abruptly from 36 mmHg to 17 mmHg and a loss of cardiac output with pulseless electrical activity was identified. The surgical intervention was immediately interrupted and advanced life support resuscitation maneuverers were initiated in the supine position. After approximately 12 minutes of resuscitation, the team identified the patient as a possible candidate for ECPR. Return to spontaneous circulation was obtained within 28 minutes but the patient remained in refractory shock with severe electric and hemodynamic instability. About 45 minutes after the cardiac arrest, the vascular cannulation was achieved and the emergency peripheral veno-arterial ECMO was initiated.

The post-cardiac arrest transthoracic echocardiography performed in the operating room revealed a dilated right ventricle with systolic dysfunction. The hemoglobin, volume status, temperature, glycemia, and electrolytes were in the normal range prior to the event. The last blood gas analysis performed before the cardiac arrest presented a slight metabolic acidosis without other significant alterations (Table 1).

**Table 1:** Blood gas analysis performed before the cardiac arrest pCO_2_: partial pressure of carbon dioxide; pO_2_: partial pressure of oxygen; SpO_2_: blood oxygen saturation; Cai: ionized calcium

Variable	Measured value	Normal range value	Unit
pH	7.312	7.35-7.45	No unit
pCO_2_	36.1	35.0-45.0	mmHg
pO_2_	193	80.0-95.0	mmHg
HCO_3_^-^	18.5	20.0-30.0	mmol/L
Base excess	-8.5	-3.0-+3.0	mmol/L
SpO_2_	99.5	>95	%
Na^+^	141	140-145	mmol/L
K^+^	4.32	3.6-5.2	mmol/L
Cl^-^	111	100-106	mmol/L
Cai	1.16	1.05-1.37	mmol/L
Lactate	0.72	<2	mmol/L

Taking into account the mentioned findings, pulmonary embolism and venous air embolism were deemed the most likely diagnoses. After the hemodynamic stabilization and suture of the surgical incision, the patient was transferred to the pediatric intensive care unit.

The post-cardiac arrest etiologic study was uneventful for alterations that support the event. The decannulation was performed after two days, and approximately 72 hours after the event, the patient was successfully extubated and aminergic support was suspended.

During hospitalization, the patient presented a good cardiac evolution and hemodynamic stability, and the post-cardiac arrest neurological status was superimposable to his pre-operative state.

## Discussion

An intraoperative refractory cardiac arrest with ECPR is undoubtedly an exceptional and challenging situation [5,6]. Despite the reported incremental trend over the last decade in ECPR statistics, the current pediatric advanced life support guidelines are still reluctant to employ this intervention. This position is mainly due to the limited evidence regarding its beneficial impact on survival rates and functional outcomes [7].

The 2020 ELSO registry found that ECPR accounts for 11.6% of all pediatric ECMO cases. Survival rates were superior in the pediatric group (42%) in comparison to the adult group (29%) [7]. Multiple studies in adults have demonstrated improved survival with ECPR compared to conventional CPR, considering survival to discharge and neurological impairment outcomes [3]. These findings have also been demonstrated in a recent systemic review and meta-analysis that reported 30% survival with favorable neurological outcomes [8]. These results seem to indicate that ECPR may play a favorable role in improving pediatric cardiac arrest outcomes.

According to the 2021 ELSO guidelines, it is imperative to establish local protocols to guide the ECPR program. If cardiac arrest is witnessed and is associated with a reversible condition, combining ECMO with high-quality conventional CPR may be considered [2]. In this setting, it is essential to define the indications and inclusion criteria to enroll the patients who would benefit from this intervention. A well-coordinated team of expert-trained professionals is essential. After the establishment of the process, the next essential step is to review the institutional experience by monitoring and evaluating outcomes to guide progress and improvement measures [3,7].

Despite its advantages, ECPR encompasses logistical and technical challenges as it requires significant medical and financial resources, which can constitute a major barrier to its local implementation. Additionally, it is not an innocuous procedure, and several complications such as hemorrhage, thrombosis, limb ischemia, infection, and irreversible neurological sequelae [3,9] have been described. Also, the low-flow time is related to successful clinical outcomes, stressing the need to reduce the duration of ECMO preparation [9]. This can cause logistic problems related to the immediate availability of the ECMO team and their respective equipment.

This case report highlights not only the determining role of ECPR in a case of pediatric refractory cardiac arrest but also its complexity in different aspects.

First, the efficient and timely application of conventional CPR was the key factor in achieving a favorable neurological outcome [7]. This efficient approach was mainly due to the clinical field where the cardiac arrest occurs - the operative room. In this specific scenario, continuous monitoring allows prompt detection and immediate assistance reducing the no-flow time [8].

Second, the balance between benefits and risks associated with the ECPR technique carried out in a surgical procedure with high hemorrhagic risk and the definition of a patient as a candidate for ECPR while conducting high-quality CPR, emphasize the complexity and challenging issues related to this case.

Third, taking into account the severe hemodynamic instability after the return to spontaneous circulation, the ECMO approach was an end-of-line measure that made it possible for the patient to survive this life-threatening complication.

Last and as a future perspective, ECMO could be used not only as a rescue but also as elective perioperative support in high-risk elective surgeries and/or in selected patients (including pediatric) with high cardiopulmonary-risk undergoing elective surgery. The use of ECMO (veno-arterial or veno-venous) as an effective support and the development of an adequate hospital referral network would allow the performance of a wide range of surgical procedures that would not otherwise be possible, especially in non-ECMO centers.

## Conclusions

ECPR encloses a potential to completely upgrade resuscitation practices, especially for pediatric patients facing cardiac arrest with reversible etiologies exposed to prolonged CPR efforts. Despite the lack of high-evidence studies in large centers about the impact of ECPR on survival and neurological outcomes, it is undeniable that it has life-saving capabilities. In tertiary centers where ECMO expertise is available, the implementation of an ECPR program could be a valuable effort to approach cardiac situations. When considering intraoperative cardiac arrest, anesthesiology and pediatric critical care departments should be involved. The local programs could also encompass the use of elective peri-operative ECMO for major surgeries and/or for high-risk patients undergoing elective surgery.
